# Prevalence of defined ultrasound findings of unknown significance at the second trimester fetal anomaly scan and their association with adverse pregnancy outcomes: the Welsh study of mothers and babies population‐based cohort

**DOI:** 10.1002/pd.4708

**Published:** 2015-11-20

**Authors:** Lisa Hurt, Melissa Wright, Frank Dunstan, Susan Thomas, Fiona Brook, Susan Morris, David Tucker, Marilyn Ann Wills, Colin Davies, Gareth John, David Fone, Shantini Paranjothy

**Affiliations:** ^1^ Institute of Primary Care and Public Health, School of MedicineCardiff University, Neuadd MeirionnyddHeath ParkCardiffUK; ^2^ Public Health Wales NHS TrustCardiffUK; ^3^ Aneurin Bevan University Health BoardCaerleonNewportUK; ^4^ Cardiff and Vale University Health BoardUniversity Hospital of WalesCardigan HouseHeath ParkCardiffUK; ^5^ National Childbirth TrustAlexandra HouseOldham TerraceLondonUK; ^6^ Cwm Taf University Health BoardYnysmeurig House, Navigation ParkAbercynonRhondda Cynon TaffUK; ^7^ NHS Wales Informatics ServiceTŷ Glan‐yr‐AfonCardiffUK

## Abstract

**Objective:**

The aim of this article was to estimate the population prevalence of seven defined ultrasound findings of uncertain significance (‘markers’) in the second trimester and the associated risk of adverse pregnancy outcomes.

**Method:**

A prospective record‐linked cohort study of 30 078 pregnant women who had second trimester anomaly scans between July 2008 and March 2011 in Wales was conducted.

**Results:**

The prevalence of markers ranged from 43.7 per 1000 singleton pregnancies for cardiac echogenic foci [95% confidence interval (CI): 38.8, 51.1] to 0.6 for mild‐to‐moderate ventriculomegaly (95% CI: 0.3, 1.0). Isolated echogenic bowel was associated with an increased risk of congenital anomalies [risk ratio (RR) 4.54, 95% CI: 2.12, 9.73] and preterm birth (RR 2.30, 95% CI: 1.08, 4.90). Isolated pelvicalyceal dilatation was associated with an increased risk of congenital anomalies (RR 3.82, 95% CI: 2.16, 6.77). Multiple markers were associated with an increased risk of congenital anomalies (RR 5.00, 95% CI: 1.35, 18.40) and preterm birth (RR 3.38, 95% CI 1.20, 9.53).

**Conclusions:**

These data are useful for counselling families and developing clinical guidance and care pathways following the detection of markers in clinical practice, particularly the need for follow‐up scans to monitor placental function and growth in pregnancies with isolated echogenic bowel, and further investigation for multiple markers. © 2015 The Authors. *Prenatal Diagnosis* published by John Wiley & Sons Ltd.

## Abstract 2

**What's already known about this topic?**

- The prevalence and clinical sequelae of defined ultrasound findings of unknown significance (‘markers’) in pregnant women at low risk of adverse pregnancy outcomes are uncertain.
- Guidance on the reporting and clinical management of markers varies between and within countries.

**What does this study adds?**

- This study provides population‐based estimates for the prevalence of markers in the general obstetric population, using data collected prior to referral to specialist fetal‐medicine centres.
- There is inter‐sonographer variation in the reporting of markers, suggesting that continuous quality assurance programmes are essential within antenatal ultrasound screening services.
- Isolated echogenic bowel and multiple markers are associated with an increased risk of congenital anomalies and preterm births, and isolated pelvicalyceal dilatation is associated with an increased risk of congenital anomalies.
- These data will be useful in counselling families and in the development of clinical guidelines and care pathways for the management of markers detected in the second trimester of pregnancy.

## Introduction

All women in the UK are offered ultrasound screening for significant fetal anomalies in the second trimester of pregnancy.^1^ The National Health Service (NHS) Fetal Anomaly Screening Programme guidance recommends screening for 11 conditions with detection rates above 50% at this scan, including anencephaly, open spina bifida and gastroschisis.^2^ ‘Defined ultrasound findings of uncertain significance’ or ‘normal variants’^3^ (referred to as ‘markers’ in this article) are also identified at this scan; these include echogenic bowel (EB), renal pelvicalyceal dilatation (PCD) and cardiac echogenic foci (CEF). Associations between markers and adverse pregnancy outcomes including intrauterine fetal death,^4^ chromosomal abnormalities^5^ and cystic fibrosis^6^ have been reported. However, many studies of markers have been conducted at specialist centres where a large proportion of pregnancies were at high‐risk of adverse outcomes.^7^ Because previous studies have used inconsistent definitions of markers or provide limited details of study population characteristics,^8^ the population prevalence and clinical sequelae of markers in women at low risk of adverse pregnancy outcomes remain uncertain. As a result, guidance on the reporting and management of markers varies both within and between countries.^1^, ^2^, ^3^, ^9^

We conducted a population‐based cohort study of unselected pregnant women receiving antenatal care in Wales, to determine the prevalence of markers and their association with adverse pregnancy outcomes (congenital abnormalities, pre‐term birth, small for gestational age (SGA) and stillbirth).

## Materials and Methods

A detailed protocol for this cohort study has been published elsewhere.^10^ All pregnant women attending routine antenatal care in six of seven Health Boards in Wales, capable of giving informed consent and having their 18 to 20 week ultrasound scan in a Welsh NHS hospital between July 2008 and March 2011, were eligible for inclusion. The majority of women joined the study during their first antenatal appointment with a healthcare professional. Informed consent was taken to record the presence of markers and use NHS numbers to link these data with routinely collected data on pregnancy outcomes.

### Ethical approval

Ethical approval for the study was given by the Multicentre Research Ethics Committee for Wales (reference 08/MRE09/17) on 16 April 2008.

### Definitions of markers

Data on seven markers (defined in Table 1) were collected during the 18 to 20 week anomaly scan. Practice in Wales at the time of the study was to report four of the following: EB, mild‐to‐moderate ventriculomegaly (VM), PCD and thickened nuchal fold (NF). Data on three additional markers were collected specifically for this study: choroid plexus cysts (CPC), CEF and short femur (SF). Information on these three markers was not included on the scan report, and study participants were not informed of their presence; this was explained to women when consent was taken.

**Table 1:** Definitions of markers used in this study and reported prevalence from previous studies

Marker	Definition	Reported prevalence at fetal anomaly scan from previous studies
Four markers routinely reported in Wales		
Echogenic bowel	Areas of increased echogenicity in the fetal bowel as bright as bone. Single or multiple loops of bowel may be identified, and it may be noted to be solid intraluminal echogenicity or occasional echogenicity of the walls only (tram line).	2–14 per 1000^4^, ^18^, ^19^
Mild‐to‐moderate ventriculomegaly	Mild‐to‐moderate ventriculomegaly is a ventricular atrial diameter, at any gestation, from 10 to 15 mm. Measurements are obtained from a transventricular axial view at the level of the glomus of the choroid plexus. The callipers were placed on the inner margins of the echogenic ventricular wall.	1 per 1000^20^
Pelvicalyceal dilatation	Fluid filled dilatation of the renal pelvis measured on axial section with an anterior–posterior diameter of 5 mm or greater (callipers to be placed on the inner AP margins of the pelvic wall). This may be unilateral or bilateral.	3–45 per 1000^4^, ^17^
Thickened nuchal fold	Thickening of the skin and the subcutaneous tissues on the posterior aspect of the fetal neck. This is best viewed in a modified biparietal diameter view to include the cavum septum pellucidum and cerebellum. Assessed by measuring the distance between the skin and occipital bone at the posterior aspect of the neck with the callipers placed on the outer edge of the bone and the outer edge of the skin. A measurement of 6 mm or greater was considered to indicate thickening before 20 + 6 weeks' gestation.	1–6 per 1000^19^, ^21^, ^22^
Three additional markers examined in this study		
Choroid plexus cysts	Small sonographically discrete fluid‐filled spaces ≥5 mm within the choroid plexus and seen on scan as black echo‐free areas. May be single, multiple, unilateral or bilateral.	6–21 per 1000^19^, ^23^
Cardiac echogenic foci	Echogenic area on the papillary muscle of either (usually left) or both of the atrioventricular valves	5–49 per 1000^19^, ^24^, ^25^
Short femur	Femur length which is below two standard deviations (3rd percentile) for gestational age when measured with the shaft of the femur parallel to the transducer. Care must be taken to ensure that the entire diaphysis of the femur is measured. If the epiphyseal cartilages are visible, they were not included in the measurement. It is assumed that the remainder of the skeleton is normal.	<50 per 1000^4^

Training on the study protocol and diagnostic criteria for each marker was given to all sonographers conducting antenatal ultrasound scans in the six participating Health Boards. Their knowledge of protocol requirements for marker measurement was assessed after training. This assessment identified over‐reporting of EB, NF and CPC, and incomplete understanding of calliper placement in the measurement of VM and the plane of measurement for PCD. Additional training on these issues was provided within each Health Board. We also established a quality assurance (QA) panel to review the ultrasound scan images of reported markers in the study.

### Ultrasound data collection

Data on the seven markers were recorded during the 18 to 20 week scan. An additional reporting screen was added to the electronic information system for radiological data storage and reporting in Wales (Radiology Information Service 2, RadIS2) to enable rapid and accurate data collection by sonographers while performing the scan. Alternative data collection arrangements were implemented in three Health Boards where RadIS2 was not yet operational, with sonographers completing a paper data collection form. At the end of recruitment, we contacted all Health Boards if scan data were missing for women who had consented to take part in the study. Where possible, data for these women were downloaded from the Health Boards' reporting systems; data on the four markers routinely reported were available from these reports, but no data on the additional three markers included in this study could be obtained from these.

The QA panel confirmed the presence of reported markers and identified false positives. A reported marker was confirmed if the panel agreed by consensus that the marker was present. They also reviewed scan images for stillbirths and babies with Down syndrome, Edward's syndrome and cystic fibrosis to ascertain whether there were any markers in those scans that had not been reported at the time of the scan. A review of all scans to assess for false negatives was not possible because of the substantial number of scans.

### Definitions of adverse pregnancy outcomes

Congenital anomalies were defined as all abnormalities included in Chapter XVII of the 10th revision of the International Statistical Classification of Diseases (codes Q00 to Q99),^11^ including congenital malformations and chromosomal abnormalities. This is consistent with the definition used in the European Surveillance of Congenital Anomalies. Data were obtained from cytogenetic results following amniocentesis or a report to the Congenital Anomaly Registry and Information Service (CARIS) in Wales up to 1 year after the birth. Preterm birth was defined as a birth before 37 weeks' gestation, and SGA was defined as below the third centile for birthweight appropriate for gestational age, stratified by sex. It was also specified *a priori* that data on stillbirths would be collected and that this outcome would be examined if there were adequate numbers. Stillbirths were defined as the *in‐utero* death of a baby after 24 completed weeks' gestation.

We used the NHS numbers of study participants to link the ultrasound scan data with data on the outcomes defined earlier from the National Community Child Health Database and CARIS. This extraction was co‐ordinated by the NHS Wales Informatics Service. An anonymised dataset for analysis was produced following linkage to scan data.

### Sample size calculation

It was anticipated that approximately 39 000 pregnant women would be eligible for recruitment. We estimated that if we achieved a 75% participation rate, and if complete ultrasound and outcome data were available for 80% of pregnancies, the final sample size would be 23 000. This would allow the prevalence of a marker with 1.0% prevalence to be estimated to within 0.1%. Assuming a marker prevalence of 1%, 23 000 pregnancies would also be adequate to detect a fivefold increase in an adverse pregnancy outcome that had a prevalence of 1%, or a twofold increase in an outcome with a prevalence of 5%, with 80% power and a 5% type 1 error rate.

### Statistical analysis

Analyses were restricted to singleton pregnancies. We used the residential postcode to assign each woman into one of the 1896 lower layer super output areas (LSOA) in Wales and hence to a social deprivation quintile derived using the Welsh Index of Multiple Deprivation,^12^ with equal counts of LSOAs in each quintile. The percentage of women in each deprivation quintile and the percentage of women with adverse pregnancy outcomes were compared between women with and without scan data, and with published data for all pregnant women in Wales, to assess how representative the study sample was of the general population.

### Calculation of marker prevalence

The prevalence of confirmed markers per 1000 singleton pregnancies in the population and 95% confidence intervals (CI) were estimated using bootstrapping with 5000 replications,^13^ to account for additional uncertainty, first, because not all scan images in which a marker had been identified were available for review and second, because those scans that were reviewed provided an estimate of the false negative rate.

### Association between markers and pregnancy outcomes

Unadjusted risk ratios with 95% CI were calculated using Stata version 13.0 (StataCorp. 2013. Stata: Release 13. Statistical Software. College Station, TX: StataCorp LP), estimating the risk of the adverse pregnancy outcomes in pregnancies where markers had been identified compared with pregnancies in which there were no markers. We performed separate analyses for isolated markers and pregnancies in which multiple markers had been identified because previous research has suggested an increased risk of adverse outcomes with multiple markers. This analysis included only confirmed markers and was therefore restricted to the study population for whom data were available on all seven markers. Due to small numbers of pregnancies with markers and adverse pregnancy outcomes, we could not stratify our analyses for other factors (such as gestational age or maternal age).

## Results

### Study sample

**Figure 1:**
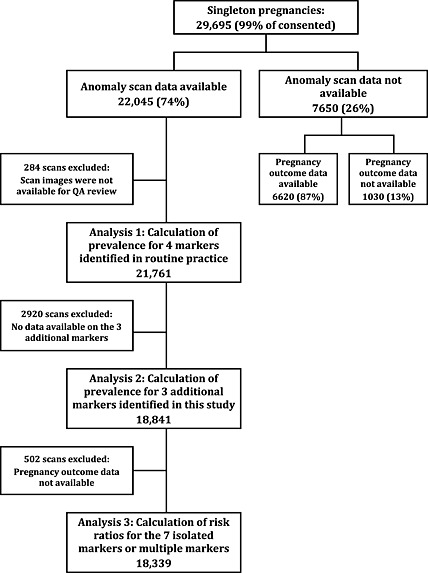
Cohort flow chart

Of the 33 252 women approached to take part, 90.5% (*n* = 30 078) consented. Of these, 29 695 had singleton pregnancies. Figure 1 shows the flow of participants through the study. Data from 22 045 scans were accessed. The prevalence of the four markers routinely reported in Wales was calculated using data from 21 761 scans (284 excluded because the images were not available for review by the QA panel). The prevalence of the three additional markers was estimated using 18 841 scans (excluding 2920 that had been obtained from the routine reporting systems of the Health Boards, which did not contain information on these additional markers). The estimates for the risk ratios were based on 18 339 scans (502 excluded as no data on adverse pregnancy outcomes were accessible for these).

### Characteristics of study women

Table 2 shows the characteristics and pregnancy outcomes of women with scan data (*n* = 22 045), without scan data (*n* = 7,651) and population data for Wales from published sources.^14^, ^15^, ^16^ The characteristics and pregnancy outcomes in the three populations were similar. Almost 85% of women with scan data were younger than 35 years of age, and 27% were classified as living in the most deprived quintile. There were 81 stillbirths in the 22 045 pregnancies with scan data (0.4%), 587 congenital anomalies (2.7%) and 1142 babies born before 37 weeks of gestation (5.4%). A higher percentage of women without scan data experienced an induced or spontaneous pregnancy loss; the timing of these lost pregnancies was not known but may explain why scan data were missing for some of these women.

**Table 2:** Characteristics and pregnancy outcomes of singleton pregnancies with scan data, eligible pregnancies without scan data and population data from published sources for Wales

Characteristic	Pregnancies with scan data	Pregnancies with no scan data	Population data from published sources^g^
	*N* (%^a^)	*N* (%^a^)	%
Maternal age^b^			
<35	18 676 (84.7)	6319 (82.6)	84.2
35+	3369 (15.3)	1327 (17.4)	15.8
Deprivation quintile^c^			
1 (least deprived)	3237 (16.4)	845 (13.0)	15.1
2	3249 (16.4)	1157 (17.8)	17.4
3	3773 (19.1)	1444 (22.2)	19.7
4	4223 (21.4)	1498 (23.1)	22.4
5 (most deprived)	5287 (26.7)	1550 (23.9)	25.5
Pregnancy outcome^d^			
Live birth	21 308 (99.4)	6247 (94.3)	99.6
Stillbirth	81 (0.4)	20 (0.3)	0.4
Induced or spontaneous pregnancy loss	48 (0.2)	358 (5.4)	
Congenital abnormalities^e^	587 (2.7)	258 (3.4)	3.3
Premature delivery (<37 weeks)^f^	1142 (5.4)	325 (5.2)	5.7

a Percentages calculated for pregnancies with available data.

b *N* = 22 045 (100%) for pregnancies with scan data and 7646 (99.9%) for pregnancies without scan data.

c *N* = 19 769 (89.7%) for pregnancies with scan data, and 6494 (84.9%) for pregnancies without scan data.

d *N* = 21 437 (97.2%) for pregnancies with scan data and 6625 (86.6%) for pregnancies without scan data.

e Calculated as a percentage of live births, stillbirths and pregnancy losses.

f Calculated as a percentage of live births.

g Data on maternal age (includes multiple pregnancies), social deprivation quintile, pregnancy outcomes and stillbirths (singleton pregnancies only) from the All Wales Perinatal Survey 2012^13^; data on major congenital anomalies from CARIS 2012^14^; and data on premature deliveries from the Office for National Statistics website information for England and Wales 2012^15^^.^

### Prevalence of markers

Table 3 shows the number and prevalence of reported markers, the prevalence range across Heath Boards, the numbers included in the QA review and the number and prevalence of confirmed markers. During the study, 1583 markers were reported; 369 of the four markers routinely reported in practice and 1214 of the three additional markers. We obtained scan images for 1295 of these for review by the QA panel; 328 (88.9%) for the markers routinely reported, and 967 (79.7%) for the other markers. Overall, one in five of the reported markers were not confirmed when the scan images were reviewed by the QA panel. The panel confirmed 242 (73.8%) of the four markers routinely reported and 784 (81.1%) of the other three markers. In addition, the QA process identified eight unreported markers (one EB, one NF, two CEF and one CPC from the review of scans with reported markers and one EB, one NF and one CEF from the images of stillbirths). No additional markers were found in the scans for babies with Down or Edward's syndrome or cystic fibrosis.

**Table 3:** Number and prevalence per 1000 singleton pregnancies of reported markers, number in quality assurance review and estimated prevalence (per 1000 singleton pregnancies) and 95% CIs for each marker

	Number of reported markers^a^	Prevalence per 1000 singleton pregnancies	Range of prevalence across Health Boards	Number (%) in quality assurance review	Number (%) confirmed	False negatives identified during quality assurance	Number of confirmed markers	Estimated prevalence per 1000 singleton pregnancies (95% CI)
*Four markers routinely reported in Wales*								
Echogenic bowel	83	3.8	2.6–5.3	78 (94.0)	55 (70.5)	2	57	4.2 (2.7, 7.0)
Mild‐to‐moderate ventriculomegaly	23	1.0	0.4–1.8	20 (87.0)	11 (55.0)	0	11	0.6 (0.3, 1.0)
Mild pelvicalyceal dilatation	221	10.0	6.6–15.9	189 (85.5)	144 (76.2)	0	144	7.6 (6.5, 8.8)
Thickened nuchal fold	42	1.9	0.2–5.1	41 (97.6)	32 (78.0)	2	34	2.9 (1.2, 5.5)
*Three additional markers examined in this study*								
Choroid plexus cysts	330	17.3	9.9–21.8	242 (73.3)	158 (65.3)	1	159	12.1 (10.1, 15.0)
Cardiac echogenic foci	858	44.9	18.5–73.7	702 (81.8)	612 (87.2)	3	615	43.7 (38.8, 51.1)
Short femur	26	1.4	0.2–3.0	23 (88.5)	14 (60.9)	0	14	0.8 (0.4, 1.3)

CI, confidence interval.

a All markers, whether isolated or not

The estimated prevalence of markers per 1000 singleton pregnancies (from commonest to rarest) was 43.7 for CEF (95% CI 38.8, 51.1); 12.1 for CPC (95% CI 10.1, 15.0); 7.6 for PCD (95% CI 6.5, 8.8); 4.2 for EB (95% CI 2.7, 7.0); 2.9 for NF (95% CI 1.2, 5.5); 0.8 for SF (95% CI 0.4, 1.3); and 0.6 for VM (95% CI 0.3, 1.0).

### Association between markers and pregnancy outcome

**Figure 2:**
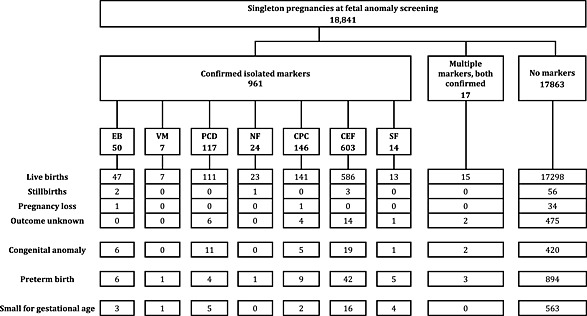
Number of confirmed isolated markers and multiple markers, and pregnancy outcomes by marker status, in singleton study pregnancies where scan data were available for all seven markers

Figure 2 shows the number of adverse pregnancy outcomes in pregnancies with isolated markers, multiple markers and no markers. The number of pregnancies with a confirmed marker and an adverse pregnancy outcome is small. For example, of the 50 pregnancies with an isolated EB, there were two stillbirths, six congenital abnormalities, six preterm deliveries and three babies with SGA. In this cohort, there were 17 pregnancies that had two markers (both confirmed by the QA panel).

In the 47 pregnancies with markers and congenital anomalies identified, there were five chromosomal abnormalities: one infant with CPC had Trisomy 18; three infants with CEF had Trisomy 21; and one infant with CPC had Trisomy 21.

Table 4 shows the risk ratios for the association of isolated markers with congenital anomalies, preterm birth and SGA for the markers, which were identified in adequate numbers (at least 50). EB was associated with an increased risk of congenital anomalies (unadjusted risk ratio [RR] 4.54, 95% CI: 2.12, 9.73) and preterm birth (RR 2.30, 95% CI: 1.08, 4.90). PCD was associated with an increased risk of congenital anomaly (RR 3.82, 95% CI: 2.16, 6.77); nine of these were anomalies of the kidneys or urinary tract. CEF were associated with an increased risk of preterm birth (RR 1.36, 95% CI: 1.01, 1.84), although the size of this association was smaller than those seen with other markers. The association between multiple markers and adverse outcomes was expected to be stronger, allowing the calculation of risk ratios based on a small number of cases. Multiple markers were associated with an increased risk of congenital anomalies (RR 5.00, 95% CI 1.35, 18.40) and preterm birth (RR 3.38, 95% CI 1.20, 9.53).

**Table 4:** Unadjusted risk ratios (95% CI) for adverse pregnancy outcomes in pregnancies with confirmed markers compared with pregnancies with no confirmed markers

	Congenital abnormalities^a^	Preterm births^b^	SGA^b^
Echogenic bowel	4.54 (2.12, 9.73)^c^	2.30 (1.08, 4.90)	1.89 (0.63, 5.67)
Mild pelvicalyceal dilatation	3.82 (2.16, 6.77)^d^	0.71 (0.27, 1.85)	1.36 (0.57, 3.21)
Choroid plexus cysts	1.44 (0.61, 3.43)	1.23 (0.65, 2.32)	0.45 (0.11, 1.80)
Cardiac echogenic foci	1.33 (0.84, 2.08)	1.36 (1.01, 1.84)	0.84 (0.52, 1.38)

CI, confidence interval; SGA, small for gestational age.

a Denominator = live births, stillbirths and pregnancy losses.

b Denominator = live births only.

c The abnormalities in the six babies with echogenic bowel were cystic fibrosis, gastroschisis, jejunal atresia, pulmonary hypoplasia, double outlet right ventricle and congenital cytomegalovirus infection.

d Nine of the eleven babies with mild pelvicalyceal dilatation were diagnosed with a renal abnormality after birth; in the other two, the diagnoses were right intra‐lobar sequestration of lung and discordant great arteries (Taussig–Bing anomaly).

Very few pregnancies with markers ended in a stillbirth and estimating a reliable risk ratio was not possible for most markers. EB was associated with an increased risk of stillbirth (RR 12.19, 95% CI: 3.06, 48.63), although this estimate should be interpreted with caution: of the two cases of EB on which the estimate is based, one was identified when the QA panel reviewed the scan of a baby that was stillborn.

## Discussion

In our population‐based study of non‐selected pregnant women, the prevalence of markers ranged from 43.7 per 1000 pregnancies for CEF to 0.6 per 1000 pregnancies for VM. One in five of the markers originally reported was not confirmed when the scan images were reviewed by a QA panel. EB and multiple markers were associated with an increased risk of congenital anomalies and preterm birth. PCD was associated with an increased risk of congenital anomalies.

This was a large study of women attending obstetric care, who were representative of pregnant women in Wales, and provides population‐based estimates for the prevalence of markers in the general obstetric population using data collected prior to referral to specialist fetal‐medicine centres. Recruitment and ultrasound data collection were embedded within the routine healthcare system, and 90.5% of women who were approached agreed to take part. The stringent QA process was a strength of the study, with scan images reviewed by an expert panel to confirm the presence of markers and ensure fidelity with the study protocol.

Scan data were only available for 74% of women who had consented to take part. This was most likely because we relied on sonographers to identify enrolled women when they attended for their scan and to access the data collection screen or complete the paper form during the scan appointment. In cases where the sonographer did not do this, we were able to access some of the missing data from the reporting system in Health Boards, although we were only able to obtain data on the four routinely reported markers using this method. The distributions of maternal age, social deprivation and pregnancy outcomes in women with and without scan data were similar. They were also comparable with routinely‐available estimates suggesting that, although these data collection methods reduced our sample size, the resulting study sample was representative of the general obstetric population in Wales.

Previous reports of the prevalence of markers at 18 to 20 week scans vary.^4^, ^17^, ^18^, ^19^, ^20^, ^21^, ^22^, ^23^, ^24^, ^25^ Our prevalence estimates are consistent with the lower end of published ranges. It is of concern that one in five of the markers originally reported in the study was classed as false positives after QA review. This finding is important, as reporting the presence of markers to parents may cause significant anxiety and can lead to the uptake of unnecessary invasive procedures.^17^

There was inter‐sonographer variability in reporting, despite clear study definitions and training at the start of the study. Variation in the reporting of markers within studies has been shown previously for PCD^26^ and VM.^27^ Differences between Health Boards were smaller for the markers routinely reported in Wales (for example, the range for EB was 2.6 to 5.3 per 1000) compared with the additional markers included in this study (for example, the range for CEF was 18.5 to 73.7 per 1000), suggesting that sonographer experience may play an important role in reporting accuracy. Audit of screening activity, continuous monitoring and feedback of screening programme parameters (including true and false positive and negative rates) should be essential activities within antenatal screening programmes using ultrasound. Evidence in the UK, however, suggests that 80% of units screening for fetal anomalies are unable to provide detection rates for various abnormalities.^28^ This highlights the need for appropriate training, re‐training and QA procedures if these markers continue to be routinely identified at antenatal screening.

Much of the previous literature had focused on the role that markers play in screening for aneuploidy. A recent meta‐analysis showed that the prevalence of markers at the second trimester scan is higher in infants with trisomy 21 than in euploid infants; however, for most isolated markers, information collected at the second trimester scan made only a small difference to the risk estimate already calculated using other screening methods (maternal age, second trimester serum biochemical testing or first trimester combined testing).^29^ Other reviews have supported this conclusion, suggesting that first trimester screening has dramatically decreased the prevalence of Down syndrome identified in the second trimester (as seen in our data)^30^ and that second trimester scans are now of most use for screening in settings when access to genetic testing is limited, when serum screening tests give inconclusive results or as ‘a noninvasive supplementary test for high‐risk women reluctant to undergo invasive diagnostic screening’.^31^

Previous studies have suggested associations between EB and other adverse pregnancy outcomes. Gestational age at birth has been shown to be significantly lower,^32^ and an increased risk of SGA and intrauterine fetal death^4^, ^32^ has been seen in pregnancies where EB has been identified. Some of our results were consistent with these findings, in that we showed an increase in preterm births and stillbirths (although our numbers for this outcome are small). Differences between our results and these studies could be explained by different definitions and different populations under study. For example, Goetzinger *et al.* 4 used birthweight <10th percentile for gestational age to identify babies with SGA, whereas we used a more restricted definition of birthweight <3rd percentile; both previous studies included intrauterine deaths from 20 weeks onwards, whereas we included deaths from 24 weeks, and Mailath‐Pokorny *et al.* 32 used data from fetal medicine centres with higher rates of adverse pregnancy outcomes compared with the general population. Goetzinger *et al.* 4 also adjusted their results for multiple potential confounders, but this did not change the magnitude of the associations seen suggesting that similar multivariate analyses may not have changed the conclusions of our analyses. The mechanism for the association between EB and these adverse pregnancy outcomes is unclear, but may involve vascular disruption leading to bowel hypo‐perfusion and ischaemia.^4^ Stillbirth, preterm birth and SGA have a multifactorial aetiology. Despite our large sample size, we were not able to account for these complexities in our analysis because of low prevalence of markers and adverse pregnancy outcomes. Nevertheless, taken together, the findings from these three studies indicate the need for monitoring pregnancies for growth and placental function following the detection of EB in the second trimester of pregnancy. Our data are also consistent with the finding that the risk of adverse outcomes is higher when two or more markers are identified^8^ and support guidance that additional investigation and follow‐up is required for these pregnancies.^1^

Associations with adverse outcomes have also been reported for markers other than EB, although information is sparse for some markers and for the long‐term implications for health during childhood. For example, a systematic review of outcomes associated with VM found that the risk of neurological abnormality diagnosed after birth was 12% to 14% (depending on the presence of infection or chromosomal abnormalities)^33^; mild pyelectasis was not associated with an increased risk of urinary tract infection during childhood in a case–control study in the Netherlands^34^; and a study in Brazil reported that 2.7% of infants with cardiac echogenic focus had cardiac anomalies, 3.7% had chromosomal abnormalities and 1.7% had cardiac defects.^35^ Further studies examining the association between specific markers and longer‐term adverse health outcomes in childhood are needed, to examine whether the associations seen in our study have implications for later health.

## Conclusion

In this study, markers were identified in more than 50 in 1000 singleton pregnancies, and associations between some markers and adverse pregnancy outcomes were seen. Our data are useful for counselling families and developing clinical guidance and care pathways following the detection of markers in clinical practice, particularly for isolated EB, and further investigation for multiple markers. Further work clarifying the implications of markers for health outcomes during childhood is needed.

## Authorship

S. P. was the principal investigator for the study. S. P., S. T., C. D., F. D. and D. F. designed the study. C. D. had the original idea for the study, provided professional advice on the obstetric ultrasound and markers, was involved with the development of RadIS2 marker screen for the study and provided oversight for the training of sonographers and the quality assurance process. F. D. provided statistical expertise, and D. F. provided epidemiological expertise. M. A. W. provided advice on the women's perspective in the study design, implementation and interpretation of data. S. T. and S. P. were responsible for the coordination and management of the study and analysed the data and produced the quality assurance report. G. J. coordinated the access to the data from different sources. M. W. was responsible for the production of a linked study database and carried out the statistical analysis, with input from F. D. S. P. and L. H. wrote the first draft of this paper. All authors contributed to the text of the paper, subsequent revisions and the production of the final version of the paper.

**WHAT'S ALREADY KNOWN ABOUT THIS TOPIC?**

- The prevalence and clinical sequelae of defined ultrasound findings of unknown significance (‘markers’) in pregnant women at low risk of adverse pregnancy outcomes are uncertain.
- Guidance on the reporting and clinical management of markers varies between and within countries.

**WHAT DOES THIS STUDY ADDS?**

- This study provides population‐based estimates for the prevalence of markers in the general obstetric population, using data collected prior to referral to specialist fetal‐medicine centres.
- There is inter‐sonographer variation in the reporting of markers, suggesting that continuous quality assurance programmes are essential within antenatal ultrasound screening services.
- Isolated echogenic bowel and multiple markers are associated with an increased risk of congenital anomalies and preterm births, and isolated pelvicalyceal dilatation is associated with an increased risk of congenital anomalies.
- These data will be useful in counselling families and in the development of clinical guidelines and care pathways for the management of markers detected in the second trimester of pregnancy.
